# Establishing the hematopoietic stem cell transplant (HSCT) in a developing country; the journey of HSCT in Semarang, Indonesia

**DOI:** 10.1038/s41409-020-0973-7

**Published:** 2020-06-17

**Authors:** Damai Santosa, Eko Adhi Pangarsa, Budi Setiawan, Ridho M. Naibaho, Daniel Rizky, Edi Dharmana, Catharina Suharti

**Affiliations:** 1 Division of Hematology/Medical Oncology, Department of Internal Medicine, Medical Faculty of Diponegoro University and Dr. Kariadi Hospital, Semarang, Indonesia; 2 Division of Hematology/Medical Oncology, Department of Internal Medicine, Medical Faculty of Mulawarman University, Parikesit General Hospital, Tenggarong, Indonesia; 3 Department of Parasitology, Medical Faculty of Diponegoro University, Semarang, Indonesia

**Keywords:** Stem-cell research, Haematological cancer

## To the Editor:

Bone marrow transplantation (BMT) is a life-saving treatment for many incurable diseases. BMT presents a valid treatment option for many congenital and acquired disorders of the hematopoietic system, including several hematological malignancies [1–4]. In BMT, failing bone marrow is supplemented by hematopoietic stem cells derived from peripheral or umbilical cord blood; therefore, BMT may also be described as hematopoietic stem cell transplantation (HSCT) [5].

Indonesia has a long involvement with BMT services. In 1987, the first allogeneic and autologous BMTs in Indonesia were performed in Semarang, Central Java by Telogorejo Hospital and Dr. Kariadi General Hospital. These transplants were performed for several cases of acute myeloid leukemia (AML), chronic myeloid leukemia (CML), and thalassemia [6]; two of the cases of AML treated in Semarang in 1987 are still in remission. Two year later in 1989, Dr. Cipto Mangunkusumo Hospital in Jakarta performed several BMTs for CML, AML, ALL cases [7]. In 1989, Dr. Kariadi General Hospital, in collaboration with Dr. Sarjito Hospital in Jogyakarta, performed BMT for a patient with AML. Then, in 1991, Hasan Sadikin Hospital in Bandung, in collaboration with Telogorejo Hospital, performed an autologous BMT for a case of AML and the patient survived for 3 years. However, none of these efforts resulted in a sustained and effective Indonesian BMT.

Semarang is the capital of Central Java Province, Indonesia. It has an area of ﻿373,78 km^2^ and a population of ~1,729,428 [8]. There are 7 public hospitals and 12 private hospitals in this city, with Dr. Kariadi Hospital being the city’s National Referral Hospital [9].

In 2012, Dr. Kariadi Hospital committed to establishing a modern BMT. Steps taken towards achieving this goal comprised of: [1] developing a Road Map for BMT services [2]; formalizing collaborative international partnerships; [3] developing appropriate clinical and laboratory infrastructure [4]; increasing human resources capacity [5]; developing clinical teamwork; and [6] fostering pharmacy services.

**Fig. 1:**
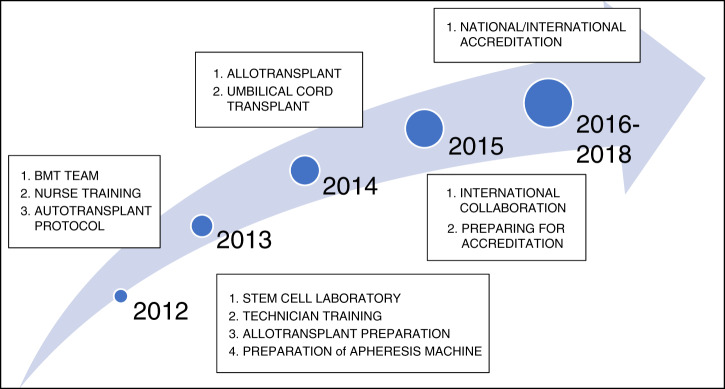
The BMT services was started in 2012 with autologous bone marrow transplant. Apharesis machine was available in 2013. In 2015, Dr. Kariadi Hospital has been accredited by Joint Committee International (JCI); and followed by BMT services in 2018.

Our team started with creating a policy framework for BMT procedures. This framework, our Road Map, is shown in Fig. 1. We began by holding BMT meetings to increase knowledge and skill for nurses, technicians, and doctors. Then, in collaboration with Singapore’s National University Hospital (NUH), one doctor, two nurses, and one analyst were sent to NUH’s BMT unit. They studied BMT-specific clinical capacities, apheresis, specific clinical nursing, and stem cell processing. One clinical pathologist was also trained in Dr. Soetomo Hospital in Surabaya in stem cell processing. Next, we invited a group known as the C2C Foundation to improve our team’s knowledge about BMT for thalassemia cases; this project involved 16 doctors and 37 nurses. Then, in 2012 and 2014, in a preceptorship program run in collaboration with the i-CML Foundation, we sent a doctor to Royal Adelaide Hospital (RAH) in South Australia to study CML management and BMT services.

Our short storage protocol calls for the suitable stem cells to be processed with RPMI, gentamicin, free preservative heparin, and held at 4 °C for a maximum of 72 h. For long term storage, we deep freeze our samples to –80 °C after processing it with DMSO 5% [10, 11]. Total Nucleated Cell and CD 34+ count is measured during harvest, post-harvest, and post thawing/before infusion. The laboratory equipments used are a Class II Biosafety Cabinet, Refrigerator (4 °C), Freezer (−20 °C), low-temperature freezer (−70 to −85 °C), and water bath. CD 34+ enumeration is calculated with a single platform by FACS Canto II [12, 13].

Our BMT team consists of a hematologist–medical oncologist, dentist, cardiologist, pulmonologist, clinical pathologist, clinical microbiologist, infectious-disease specialist, psychologist, and psychiatrist. Every Wednesday we hold a BMT meeting to share knowledge, skill, support management, and clinical approach. Our clinical pharmacist supplies and evaluates the adverse event of medications.

Today we have ten positive pressure rooms with HEPA filters; six rooms are located in the Kasuari Oncology Cancer Center and four rooms are in the Rajawali building. By 2018, we had already performed 16 HSCTs, including 2 allogeneic transplants. Diagnoses of the subjects receiving HSCT included AML, multiple myeloma, relapsed non-Hodgkin Lymphoma (NHL), relapsed AML, and myelodysplastic syndrome (MDS). The donor source of the allogeneic transplants were siblings. Baseline patient characteristics and transplant data of our autologous and allogeneic patients are listed in Table 1.

**Table 1:** Baseline patient characteristics and transplant data of our autologous and allogeneic patients.

Characteristics	Autologous	Allogeneic
Total	14	2
Age, median (IQR)	43.5 (18–62)	31.3 (24–39)
Gender: Male/Female	9/5	0/2
Indication	Multiple myeloma, AML, Relapsed NHL	MDS Relapsed AML
Stem cell source	Peripheral blood Bone marrow	Peripheral blood
Stem cell (CD34+) dose (median, IQR)	3.21 (1.25–6.80)	3.58 (2.91–4.26)
Donor	Not applicable	All siblings
HLA matching		Fully match

*HLA* human leukocyte antigen, *IQR* interquartile range, *NHL* non-Hodgkin lymphoma, *AML* acute myeloid leukemia.

No severe adverse events were reported at thawing (for example, clump formation). Grade 1 transient reactions, according to Common Terminology Criteria for Adverse Events (CTCAE criteria) [14] were recorded during reinfusion of cryopreserved cells (flushing, headache, hypertension, hemoglobinuria, bradycardia, shivering, and fever; the incidence of these reactions were 7, 6, 5, 5, 2, 2, and 1, respectively). The allogeneic transplant was performed in two patients with the full match from their siblings. Two autologous (two cases of relapsed NHL) and two allogeneic (relapsed AML and MDS) patients died during the procedure due to transplant-related mortality [15].

Every patient except one achieved neutrophil and platelet engraftment, at a median of day + 13 and day + 15, respectively. The most common serious adverse events during the transplantation were mucositis, nausea vomitus, neutropenia, sepsis, diarrhea, hair loss, electrolyte imbalance, anemia, thrombocytopenia, and febrile neutropenia. Hyperpigmentation developed at patient with myeloma given high dose melphalan.

Prophylaxis with ciprofloxacin, acyclovir, ganciclovir, fluconazole, and trimethoprim were given according to the protocol to reduce the risk of infection. The blood cultures in our patients with febrile neutropenia were generally found to be negative for aerobes, anaerobes, and fungal microorganisms; however, positive cultures for *Escherichia coli*, *Pseudomonas aeruginosa*, and *Klebsiella pneumonia* were achieved. Patients with febrile neutropenia were treated according to the guidelines [16, 17]. The overall survival rates 3 months, 6 months, and 1-year post-treatment were 56.25%, 56.25%, and 50%, respectively.

Supportive treatments, including blood component transfusion, were given according to guidelines, and all blood products were treated with a single dose of radiation at 15–25 Gy. Patients being treated according to our BMT protocol are treated with filtered and irradiated blood products. Irradiated products are cytomegalovirus-safe, have a reduced risk of nonhemolytic febrile transfusion and alloimmunization, and have a reduced incidence of transfusion-associated graft vs host disease in at-risk individuals [18–21].

We have pioneered a bone marrow donor’s program called “Transformer”, which consists of 30 volunteers who have been HLA typed. We also receive support from nonprofit organizations including the Hematology and Thalassemia Foundation and the Indonesian Cancer Foundation. To improve our BMT program further, we need to utilize every support we can. Of particular importance is continuing to strengthen our donor program, through means including the training of our human resources, developing our systems further, website design, and increasing the number of our volunteers [22, 23].

Indonesia’s Ministry of Health has developed several health funding initiatives, including the National Health Insurance Program, the Healthcare and Social Security Agency, and the Indonesian Health Card. National Health Insurance covers over 81% of Indonesian citizens. Our BMT service is already listed in the national healthcare insurance system with a limited budget (±7005.60 USD)[24]. We need to advocate and collaborate with the Ministry of Health in the future to improve and resolve this financial constraint.
